# Longitudinal mediation effects of activity meaning on the association between activity performance and quality of life among older adults with disabilities

**DOI:** 10.1186/s12877-023-04451-7

**Published:** 2023-11-11

**Authors:** Shiau-Fang Chao, Chin-Yi Su, Ming-Fang Chang

**Affiliations:** Department of Social Work, National Taiwan University, No 1, Section 4, Roosevelt Road, Daan District, Taipei, 106319 Taiwan

**Keywords:** Meaning, Activity performance, Quality of life, Disability, Longitudinal

## Abstract

**Background:**

Physical limitations may hinder older adults with physical disabilities’ capability to perform various activities, which can affect their quality of life (QOL). Accomplishing meaningful activities may mitigate the impact of limited activity performance on their QOL. This longitudinal study aims to investigate how activity meaning mediates the relationship between activity performance and QOL among older adults with disabilities.

**Methods:**

Data for this longitudinal study was collected from 813 community-dwelling older adults aged 60 and above who had physical disabilities, over a two year interval. Path analysis was used to examine the cross-sectional and longitudinal mediation effects from activity performance, through activity meaning, to QOL.

**Results:**

At the same wave, high IADL performance or social activity performance, and high QOL was indirectly associated through high IADL meaning or social meaning. As for longitudinal association, high T1 IADL performance was associated with better T2 QOL through high T1 and T2 IADL meaning. Similarly, high T1 social activity performance also contributed to T2 QOL through high T1 and T2 social activity meaning. Additionally, social activity performance exhibited higher influence on QOL than that of IADL.

**Conclusions:**

Both IADL and social activities have distinct impacts on the QOL of older adults with disabilities. To improve the current and future QOL of older adults with disabilities, professionals must prioritize their involvement in the most meaningful activities while being sensitive to and supportive of their preferences and valued lifestyles.

## Background

Taiwan’s aging population has seen significant growth in recent years. In 2018, Taiwan officially became an aged society, with 14.5% of its population over the age of 65. Projections suggest that Taiwan will reach hyper-aged status by 2026, with the older adult population accounting for 20% of the total population. Using an estimated Activities of Daily Living (ADL) disability rate of 12.7%, the number of older adults with disabilities was 43.6 thousand in 2018, 48 thousand in 2020, and is expected to reach 62 thousand by 2026 in Taiwan [1].

The Activity Theory posits that older adults can achieve higher levels of fulfillment and happiness by actively participating in social interactions and remaining physically active [2]. The Continuity Theory suggests that older adults can achieve an optimal aging status by preserving activities that draw upon past experiences to foster continuity in psychological characteristics, social behavior, and circumstances [2]. Nevertheless, Atchley (1989) [2] noted that these theories do not take into account the potential changes in physical condition that can occur with aging. When facing pathological aging or declined health condition, the approaches suggested by these theories may become impractical, rendering them less suitable for older adults with disabilities. As a result of physical limitations, older adults with disabilities often spend more time indoors and participate in fewer activities, such as daily activities (e.g., housework and self-care) or social activities, compared to their healthy counterparts [3–5]. However, studies have demonstrated that participating in activities exerts a stronger influence on the health-related quality of life (QOL) of older adults with disabilities when compared to those without disabilities [6].

Katz et al. suggested that disability can be assessed based on an individual’s limited ability or opportunities to perform activities across three categories. These categories include obligatory activities, which refers to activities necessary for survival (e.g., sleep or take care of basic needs); committed activities, which are associated with fulfilling primary productive social roles (e.g., IADL or taking care of family members); and finally, discretionary activities (e.g., visiting with friends or family members in their home, participation in leisure activities outside an individual’s home, or traveling), which encompass activities related to socializing, relaxation, and pleasure [7, 8]. Among these three categories, committed activities and discretionary activities reflects more complex functioning and are more likely to be affected by pathology and impairment. As a results, limitations in these complex activities are a more sensitive measure to the influence of disability on an individual’s perception QOL than those only involved basic needs (i.e., obligatory activity) [7]. In addition, although both IADL and social activities are considered more complex life activities, their positive relationship with QOL may arise from fulfilling different psychological needs. The former could be linked to individual competence and autonomy, while the latter could foster a sense of relatedness [9].

Most existing studies have focused on objective indicators, such as the frequency of activity participation [10–12]. Insufficient research has been conducted on changes in activity performance among older adults with disabilities over time. For instance, Lefrancois et al. [3] examined changes in participation in valued activities over one year and found that a decline in the ability to perform IADL led to a reduction in participation in diverse activity categories, including physical, social, cognitive, and emotional activities. Katz et al. [8] additionally proposed that individuals with more severe disabilities encountered a greater increase in difficulty in engaging in valued activities over a one-year period. Of the three types of valued activities defined earlier, committed activities (e.g., IADL or taking care of family members) and discretionary activities (e.g., visit with friends or family members in their home, participation in leisure activities outside an individual’s home, or traveling) were more affected by declining physical status than obligatory activities (e.g., sleep or take care of basic needs).

In addition, the meaning of activities may have a pivotal role in the connection between activity performance and QOL. Activity meaning reflects an individual’s abilities, preferences, habits, and social roles. Older adults may have varying attitudes towards the same activity, and the same activity may hold different meanings for an individual over time as their environment and health condition change [13, 14]. According to the Social-emotional selectivity theory (SST), individuals perceive their future time as limited as they grow older. This perception of limited time influences their goal prioritization, leading them to prioritize emotionally meaningful objectives and invest more in emotionally fulfilling experiences [15, 16]. Herein, this assumption may apply to the performance of both instrumental activities of daily living (IADLs), which determine an older individual’s ability to live independently in the community and impact their fulfillment of primary social roles, as well as social activities, which demonstrate their degree of active involvement in society. The sequence of choosing to retain or abandon performing specific IADL tasks or social activities for older adults with disabilities, if they need to make choices under physical or mental constraints, can vary depending on their subjective meaning, importance, and emotional rewards associated with each task or activity [17]. Additionally, while the deteriorating physical condition and decreased performance in activities may undermine the self-worth of older adults with disabilities, engaging in meaningful activities can help them maintain a positive or establish a new sense of self-identity, thereby preserving their QOL [18].

Empirical research has also not adequately explored the subjective activity experiences and QOL of older individuals with disabilities, especially the longitudinal relationship [19]. To the best of our knowledge, only one study by Levasseur et al. [20] has examined the longitudinal association between engagement in meaningful activity and QOL in older adults with disabilities. The study utilized the Assessment of Life Habits (Life-H 3.0), developed by Fougeyrollas et al. (1998). The assessment has 69 items in 12 life domains: six for daily activities (e.g., nutrition, personal care, communication, mobility) and six for social role participation (e.g., interpersonal relationships, community life, volunteer activities, recreation) [20]. Levasseur et al. [20] found that greater satisfaction in the participation of social role-related activities and daily activities at baseline was related to higher QOL at a two-year follow-up. This study also indicated that satisfaction with participation in social activities at baseline was a stronger predictor of QOL at follow-up than satisfaction with participation in daily activities at baseline, suggesting that different types of subjective activity appraisal may have varying effects on QOL. However, the study’s small sample size (n = 49) restricted the use of more rigorous analytical methods to test hypothesized longitudinal relationships.

**Fig. 1:**
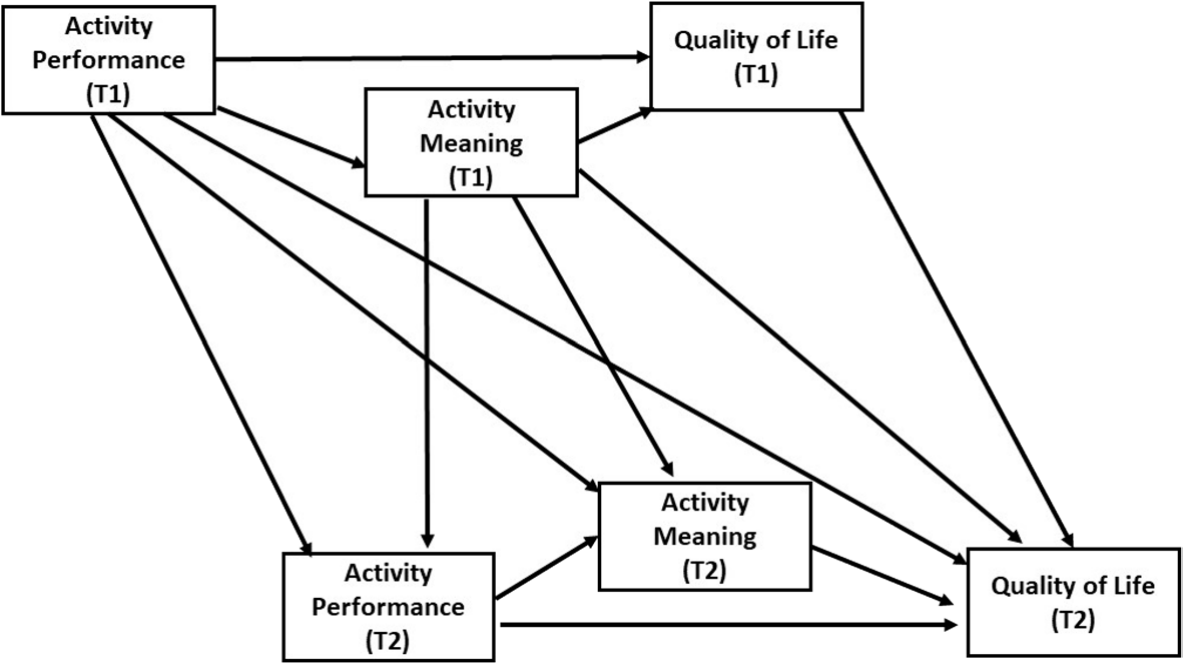
Conceptual framework

Integrating the previously mentioned literature on gerontology and disability, the aim of this study is to investigate how the performance of more complex life activities, that is, IADL (e.g., meal preparation, housework, or shopping for daily needs) and social activities (e.g., visiting relatives, volunteering, or engaging in outdoor activities), may exert cross-sectional and longitudinal influences on the QOL of older adults with physical disabilities. Additionally, the study aims to explore how these associations are mediated by the meaning of IADL or social activity. The performance of IADLs determines an individual’s ability to live independently in the community and also affects their ability to fulfill primary social roles. Social activities indicate the degree to which individuals are involved in a broader scope of society, taking into account functional limitations. The investigation of IADLs and social activities offers a more comprehensive understanding of the overall life situation of older adults with disabilities, rather than solely focusing on their basic needs. Drawing from the SST theory and prior research, it is suggested that subjective assessment of activity participation, rather than objective indicators, may have a critical impact on the QOL of older adults with disabilities. Therefore, the following hypotheses were tested (see Fig. 1): (1) At both T1 and T2, activity performance and QOL are mediated by activity meaning at the same wave; (2) Better T1 activity performance predicts higher T2 QOL through higher T1 activity meaning and higher T2 activity meaning; (3) The cross-sectional and longitudinal mediation association among activity performance, activity meaning, and QOL may differ according to activity type, particularly IADL and social activities. Even as the level of disability increases, the results of this study offer the potential to maintain or enhance the QOL of older individuals with disabilities by sustaining or improving their engagement in meaningful activities.

## Methods

### Sample

This longitudinal study involved 813 older adults aged 60 and over with physical disabilities who lived in the community who completed both T1 and T2 surveys. The eligibility criteria were that participants must have lived in the community for more than three months, be able to understand and answer survey questions, and be unable to perform at least one ADL or IADL. The T1 survey was conducted between April and July 2018, and the follow-up (T2) survey was conducted between April and July 2020. At T1, the researchers first invited local long-term care service providers and volunteers who served community-dwelling older adults with disabilities (such as home care agencies, community care stations, or adult day care centers) to participate in data collection. These providers and volunteers then conveyed the invitation to the older individuals they served, who were asked to participate in face-to-face interviews voluntarily. Interviews were conducted at the participants’ homes, community care stations, or adult day care centers, depending on the convenience of the participants.

A total of 1,314 older adults with disabilities completed T1 survey and 831 of them completed the follow-up questionnaire (T2). Out of the 483 dropouts, 243 refused to participate, lost contact, or had moved away, while 132 passed away, 73 were placed in long-term care facilities, and 35 couldn’t comprehend the questions. The other 18 participants were then excluded because they did not complete the activity scale. At last, a total of 813 participants were included. Differences between participants who completed the follow-up and those who dropped out were examined using Chi-square or t-tests. The findings revealed that older age, male, and greater ADL disability at T1 were associated with a higher likelihood of dropping out by T2. The study was approved by the Institutional Review Board at National Taiwan University (No. 201805HS004).

### Measures

#### Activity performance

This study refers activity as a diverse array of activities that individuals find meaningful or pleasurable, and extends beyond activities that are essential for survival or self-sufficiency [7]. The activities utilized in this study were adopted from Katz et al.‘s [8] valued life activity scale and the 12 life domains of Life-H 3.0 developed by Fougeyrollas et al. [20], both mainly used two types of activities, IADL and social activity, to measure activity performance. Both scales have been confirmed as valid and reliable measures for life habits or life activities in general or older persons with disabilities [7, 21]. In this study, IADL performance was assessed using six items, including preparing meals, taking medication, doing housework, shopping for daily necessities, managing finances, and making phone calls. Social activity performance was evaluated using ten items, including visiting friends or relatives, inviting others to one’s home, dining out, taking overnight trips, providing care for others, doing volunteer work, joining clubs, attending religious activities, engaging in outdoor activities, and participating in regular exercise classes. Participants rated their capability in performing IADLs or social activities on a scale of 0 (unable to do) to 3 (no difficulty). The scores for the individual items in IADLs or social activities were summed, respectively (IADL: range 0–18; social activities: range 0–30), and a higher total score indicated better IADL or social activity performance (IADL: α = 0.881 for T1 and α = 0.908 for T2; social activity: α = 0.960 for T1 and α = 0.963 for T2).

#### Activity meaning

The construct of activity meaning pertained to both IADL and social activity types. Participants were asked to rate the level of meaningfulness for each activity type. Activity meaning was scored on a 1–5 scale, with 1 indicating a complete lack of meaningfulness and 5 indicating a high degree of meaningfulness. Total scores for IADL (range 6–30) and social activity meaning (range 10–50) were calculated separately, with higher scores reflecting greater levels of meaning attached to the respective activities (IADL: α = 0.868 for T1 and α = 0.913 for T2; social activity: α = 0.867 for T1 and α = 0.895 for T2).

#### Quality of life

QOL denotes the subjective assessment of an individual’s emotional, physical, and social well-being, reflecting the extent to which the individual perceives their current life as meeting their own and societal expectations [22]. The WHOQOL-AGE scale was utilized to evaluate the QOL of participants, which has been validated for use with non-institutionalized older adults [23]. The QOL scale is composed of 13 indicators, with responses ranging from 1 (very dissatisfied) to 5 (very satisfied), to measure various aspects of the participant’s health, physical functioning, interpersonal relationships, housing, use of time, and control over things. The total score is computed following Caballero et al.‘s [23] recommendation, with a higher score indicating a better QOL (α = 0.897 for T1; α = 0.907 for T2).

#### Control variables

Four demographic variables at T1, including age (*1 = 60–74 years old; 2 = 75–84 years old; 3 = 85 years old and above*), gender (*1 = male; 2 = female*), education level (*1 = elementary school and below; 2 = junior high school and above*), and monthly income (*1 = below NT$5,999; 2 = NT$6,000 to 11,999; 3 = NT$12,000 to 17,999; 4 = NT$18,000 and above*), as well as T1 ADL scores, were included as control variables. ADL scores was assessed by summing the performance ratings for six tasks, including bathing, dressing, toileting, transferring (e.g., moving from bed to chair), continence management, and feeding oneself [24]. Each task was assigned a score between 0 (*unable to perform*) and 3 (*no difficulty*), with higher scores indicating greater performance in ADL tasks. Existing literature suggests that older age, female gender, lower ADL ability, lower education, and monthly income are associated with higher functional limitations, lower valued activity performance, and lower QOL. As these five variables have the potential to influence an older individual’s access to resources and ability to participate in activities, which can ultimately impact their QOL [6, 25–27].

### Analytic plan

SPSS Statistics 22.0 and Amos 22.0 were used for data coding and analysis. Descriptive analysis were firstly conducted to explore the sample characteristics. As this study involves two repeated measurements on the same sample, paired t-tests were used to examine whether there were any significant differences in the means of key variables of interest between T1 and T2. Moreover, the normality of the distribution for key variables of interest was examined. The skewness and kurtosis values for IADL and social activities performance and meaning, as well as QOL at both T1 and T2, were close to or less than ± 1. Therefore, the distributions of the data variables were considered to be normal. Thus, Pearson product-moment correlation analysis was used to examine the bivariate relationships among the main variables in this study [28].

To establish the factor structure of the 16 activities under study, exploratory factor analysis (EFA), utilizing principal component estimation, was then employed [29]. The 16 activities were categorized as IADL and social activities according to the EFA results. Path analysis was then conducted to examine the cross-sectional (Hypothesis 1) and longitudinal (Hypothesis 2) mediation effect from activity performance, through activity meaning, to QOL. The aforementioned path analysis was performed separately for IADL and social activities to investigate whether the mediation effects of activity meaning on the relationship between IADL/social activities and QOL differ depending on the activity type (Hypothesis 3). In all path analysis models, the correlations of all control variables with T1 and T2 IADL or social activity performance, as well as T1 and T2 QOL were considered. Bootstrap results by AMOS were used to test the significance of the indirect effect, with 2,000 bootstrap re-samplings used to retrieve the 95% confidence interval.

## Results

### Demographic characteristics, changes in disability, and correlation of variables

Table 1 displays the demographics of the 813 participants, revealing that the majority were female (70.5%), aged 75–84 in T1 (43.1%), and had an elementary education level or below (74.5%). Paired sample t-tests revealed that there is no significant difference in the degree of ADL disability between T1 and T2. Paired sample t-tests also indicated a significant decline in mean scores for all main variables, including IADL performance, social activity performance, IADL meaning, social activity meaning, and QOL, from T1 to T2.

**Table 1:** Characteristics of the sample

Variables	T1 (*N* = 813)	T2 (*N* = 813)
	Mean (SD) or %	Mean (SD) or %
**Gender**		
Male	29.5%	
Female	70.5%	
**Age**		
60–74 years old	32.6%	
75–84 years old	43.1%	
85 years old and above	24.4%	
**Education level**		
Elementary and below	74.5%	
Junior high and above	25.5%	
**Monthly income**		
5,999 NTD and below	27.1%	22.4%
6,000–11,999 NTD	39.1%	39.5%
12,000–17,999 NTD	20.7%	18.1%
18,000 NTD and above	13.2%	20.0%
**ADL score** (0–18)	14.46(3.68)	14.29(4.42)
**Activity performance**		
IADL performance (0–18)	12.63(4.63)	12.00(5.47)
Social activity performance (0–30)	13.20(8.60)	12.04(8.96)
**Activity Meaning**		
IADL meaning (6–30)	21.04(5.09)	19.83(6.45)
Social activity meaning (10–50)	27.92(9.66)	25.37(9.63)
**Quality of life** (15–65)	42.92(8.36)	42.07(8.82)

*ADL* Activities of daily living; *IADL* Instrumental activities of daily living; *NTD* New Taiwan Dollar

Among the 813 participants included, the three most frequently encountered ADL difficulties at T1 and their respective percentages were walking (60.0%), bathing (50.2%), and transferring (49.5%). The three least difficult ADL tasks were dressing (45.2%), toileting (40.7%), and eating (21%). As for IADL tasks, the most commonly reported difficulties were associated with doing household chores (69.3%), meal preparation (67.3%), and going shopping (65.4%). The three least difficult IADL tasks were managing money (40.8%), making phone calls (37.3%), and taking medication (36.6%). From T1 to T2, participants experienced significantly increasing difficulties in three ADL tasks, including dressing, toileting, and bathing, as well as in all IADL tasks, except for making phone calls (results not shown).

Table 2 presents the correlation matrix for the main variables in this study, demonstrating that both IADL and social activity performance, IADL and social activity meaning, and QOL were positively associated with each other, both cross-sectionally and longitudinally.

**Table 2:** Zero-order correlations among key model variables

	1.	2.	3.	4.	5.	6.	7.	8.	9.	10.
1. IADL Performance (T1)	-									
2. IADL Performance (T2)	0.671**	-								
3. IADL Meaning (T1)	0.226**	0.209**	-							
4. IADL Meaning (T2)	0.282**	0.415**	0.265**	-						
5. Social Activity Performance (T1)	0.618**	0.547**	0.197**	0.244**	-					
6. Social Activity Performance (T2)	0.533**	0.675**	0.119**	0.351**	0.670**	-				
7. Social Activity Meaning (T1)	0.275**	0.225**	0.473**	0.212**	0.469**	0.316**	-			
8. Social Activity Meaning (T2)	0.292**	0.391**	0.170**	0.548**	0.398**	0.569**	0.345**	-		
9. Quality of Life (T1)	0.499**	0.416**	0.287**	0.247**	0.545**	0.448**	0.379**	0.295**	-	
10. Quality of Life (T2)	0.376**	0.505**	0.148**	0.334**	0.430**	0.533**	0.255**	0.400**	0.527**	-

*IADL* Instrumental activities of daily living

*p ≦ 0.05, **p ≦ 0.01, *** p ≦ 0.001

### Exploratory factor analysis (EFA) of activity performance

As the 16 activities used in this study were adopted from previously developed valued life activity and life habits scales, which were originally developed using Western samples, it is necessary to conduct an EFA to examine the factor structure of these 16 activities among older adults with disabilities in Taiwan before proceeding with activity classification. The EFA results indicated that a two-factor structure produced a satisfactory model fit (*χ*^*2*^_(103)_ = 1437.2, *p* < .001; RMSEA = 0.099; CFI = 0.906). Of the 16 activities, six items and 10 items had factor loadings greater than 0.6 on the IADL and social activity factor, respectively (as shown in the measures “*Activity performance*” section). This two-factor structure of activity performance explained 72.82% of variability.

### Testing the IADL activity model

**Fig. 2:**
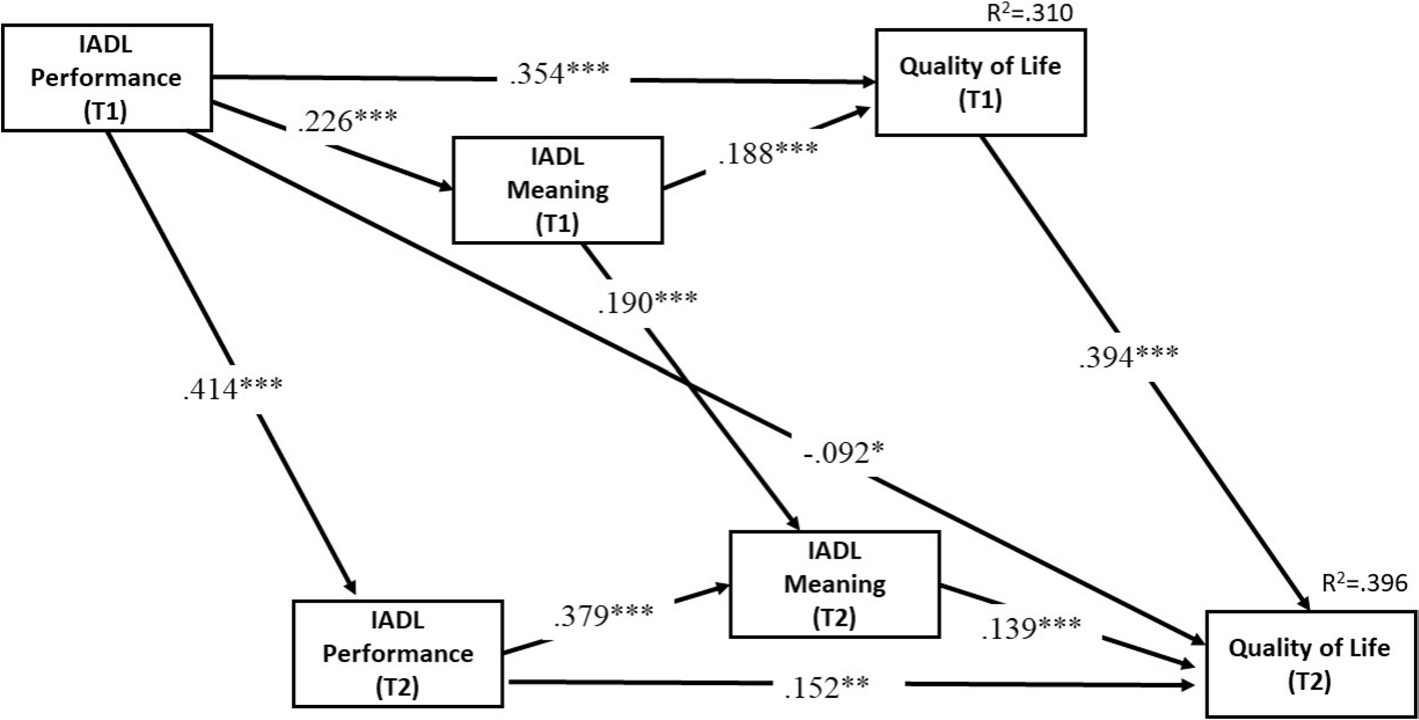
Standardized regression coefficients of IADL activity path analysis with covariates controlled (only significant paths shown).  Note: IADL = Instrumental activities of daily living; Covariates = age, gender, education, income, activities of daily living (ADL) score.  *p ≦ 0.05, **p ≦ 0.01, *** *p* ≦ 0.001

Table 3 presents the standardized direct and indirect effects of IADL performance on QOL, while Fig. 2 displays the significant standardized regression coefficients of the IADL activity path. The model exhibited an acceptable fit with the data (*χ*^*2*^_(32)_ = 157.327, *p* < .0.001; RMSEA = 0.070; CFI = 0.964).

**Table 3:** Standardized direct and indirect effects of T1 and T2 IADL performance on T1 and T2 QOL

	Outcome					
	QOL (T1)			QOL (T2)		
	Direct	Indirect	Total	Direct	Indirect	Total
T1						
IADL Performance	0.354***	0.043***	0.396***	− 0.092*	0.235***	0.143***
IADL Meaning	0.188***	-	-	− 0.047	0.109***	0.061
T2						
IADL Performance	-	-	-	0.152**	0.053***	0.205***
IADL Meaning	-	-	-	0.139***	-	-

*IADL* Instrumental activities of daily living; *QOL* Quality of Life

*p ≦ 0.05, **p ≦ 0.01, *** *p* ≦ 0.001

### Cross-sectional effects of IADL performance and IADL meaning on QOL

Figure 2 illustrates that T1 IADL performance and T1 IADL meaning accounted for 31.0% of the variance in T1 QOL. As demonstrated in both Fig. 2; Table 2, regardless of T1 or T2, IADL performance had a direct effect on QOL at the same wave (T1 β = 0.354, *p* < .001; T2 β = 0.152, *p* < .001), as did IADL meaning on QOL (T1 β = 0.363, *p* < .001; T2 β = 0.210, *p* < .001). In comparison of the standardized parameters, IADL performance exhibited a stronger cross-sectional relationship with QOL than IADL meaning did at both T1 and T2. Moreover, Table 2 reveals that IADL performance was also indirectly associated with QOL through IADL meaning at the same wave, in both T1 and T2 (T1 β = 0.043, *p* < .001; T2 β = 0.053, *p* < .001). By demonstrating the mediation effect of IADL meaning in the link between IADL performance and QOL at the same wave, the evidence supports hypothesis 1.

### Longitudinal effects of IADL performance and IADL meaning on QOL

Regarding the longitudinal direct effects, Fig. 2 demonstrates that T1 IADL performance (β = 0.414, *p* < .001), IADL meaning (β = 0.190, *p* < .001), and QOL (β = 0.394, *p *< .001) were positively related to their respective T2 variables. As shown in Fig. 2; Table 3, T1 IADL performance had a positive indirect effect (β = − 0.235, *p* < .001) on T2 QOL through five paths, with a total positive indirect effect of 0.143 (*p* < .001): (1) T1 QOL -> T2 QOL; (2) T1 IADL meaning -> T1 QOL -> T2 QOL; (3) T1 IADL meaning -> T2 IADL meaning -> T2 QOL; (4) T2 IADL performance -> T2 IADL meaning -> T2 QOL; (5) T2 IADL performance -> T2 QOL. Research hypothesis 2 was confirmed, as both T1 and T2 IADL meaning played a significant intervening role in the relationship between T1 IADL performance and T2 QOL.

### Testing the social activity model

**Fig. 3:**
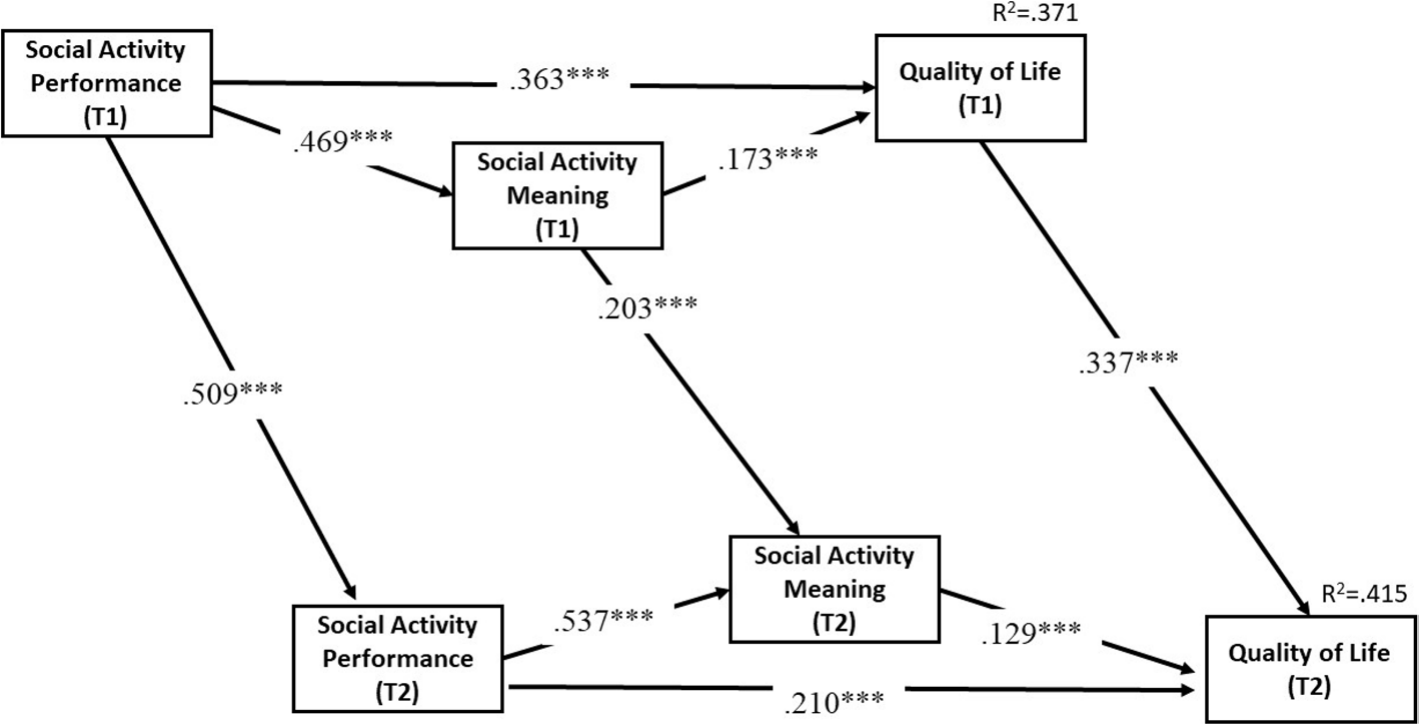
Standardized regression coefficients of Social Activity path analysis with covariates controlled (only significant paths shown).  Note: Covariates = age, gender, education, income, activities of daily living (ADL) score.  *p ≦ 0.05, **p ≦ 0.01, *** *p* ≦ 0.001

The regression coefficients for the significant social activity paths are presented in Fig. 3. The model achieved a satisfactory fit with the data (*χ*^*2*^_(32)_ = 106.119, *p* < .001; RMSEA = 0.053; CFI = 0.977), and the parameters for both cross-sectional and longitudinal path coefficients are shown in Table 4.

**Table 4:** Impact of Social Activity Performance on QOL: Standardized direct and indirect effects

	Outcome					
	QOL(T1)			QOL(T2)		
	Direct	Indirect	Total	Direct	Indirect	Total
T1						
Social Act. Performance	0.363***	0.081***	0.444***	− 0.068	0.298***	0.230***
Social Act. Meaning	0.173***	-	-	− 0.001	0.090***	0.089
T2						
Social Act. Performance	-	-	-	0.210***	0.069***	0.279***
Social Act. Meaning	-	-	-	0.129***	-	-

*IADL* Instrumental activities of daily living; *Act.* Activity; *QOL* Quality of Life

*p ≦ 0.05, **p ≦ 0.01, ***p ≦ 0.001

### Cross-sectional effects of social activity performance, social activity meaning and QOL

Based on Fig. 3, social activity performance and meaning at T1 accounted for 37.1% of the variance in T1 QOL. The figure and Table 4 show that both social activity performance (T1 β = 0.363, *p* < .001; T2 β = 0.210, *p* < .001) and social activity meaning (T1 β = 0.173, *p* < .001; T2 β = 0.129, *p* < .001) had direct effects on QOL at the same wave. The standardized parameters revealed that social activity performance also had a stronger positive effect on QOL than social activity meaning, which was consistent with the findings from the IADL paths. Additionally, the results in Table 4 indicated that social activity meaning mediated the relationship between social activity performance and QOL (T1 β = 0.081, *p* < .001; T2 β = 0.069, *p* < .001) at the same wave, also providing further support for research hypothesis 1.

### Longitudinal effects of social activity performance, social activity meaning and QOL

Table 4 demonstrates that T1 social activity performance, social activity meaning, and T1 QOL had positive direct effects on their respective 0T2 variables, similar to the findings observed in the IADL paths. Regarding longitudinal indirect effects, the analyses demonstrated a significant indirect effect (β = 0.298, *p* < .001) of T1 social activity performance on T2 QOL through five paths that were similar to those observed for IADL. The positive indirect impact of 0.298 (*p* < .001) was observed in all five of these indirect pathways. Given the significant mediating influence of both T1 and T2 social activity meaning on the relationship between T1 social activity performance and T2 QOL, the second research hypothesis was confirmed.

### Differences in the cross-sectional and longitudinal effects of IADL and social activity on QOL

The comparison of Tables 3 and 4 indicates that social activity performance yielded a greater cross-sectional impact on QOL than IADL, especially at T2. The standardized direct effect of T1 social activity performance on T1 QOL (β = 0.363, *p* < .001) was slightly greater than that of T1 IADL performance on T1 QOL (β = 0.354, *p* < .001). Nevertheless, T2 social activity performance exhibited greater direct effects on T2 QOL (β = 0.210, *p* < .001) than that of T2 IADL performance on T2 QOL (β = 0.152, *p* < .001). Additionally, Tables 3 and 4 also reveal that the indirect effects of T1 social activity performance on both T1 and T2 QOL (T1 β = 0.081, *p* < .001; T2 β = 0.298, *p* < .001) through intervening variables were also stronger than those of T1 IADL performance (T1 β = 0.043, *p* < .001; T2 β = 0.235, *p* < .001). These findings are consistent with the third research hypothesis, which suggests that the connections between T1 activity performance through T1 or/and T2 activity meaning to T1 or/and T2 QOL differ based on the type of activity.

## Discussion

This study found that IADL or social activity performance had a direct positive effect on QOL at the same wave. The positive association between IADL or social activity performance and QOL was mediated by IADL or social activity meaning at the same wave. Furthermore, better IADL or social activity performance at T1 was also related to T2 QOL through their associations with higher IADL or social activity meaning at both T1 and T2, as well as better IADL or social activity performance at T2. Additionally, social activity performance at both T1 and T2 yielded a stronger influence on QOL at the same wave than IADL, and the variations in T2 QOL were better explained by the performance and meaning of social activities at both T1 and T2, compared to IADL.

Consistent with the first hypothesis, the cross-sectional analysis conducted on both T1 and T2 data confirmed the mediation role of activity meaning between activity performance and QOL for both IADL and social activities. In other words, older adults with disabilities who perform well in either IADL or social activities tend to attribute high meaning to these activities, which in turn leads to higher QOL. The results support the SST assumption [15], suggesting that despite limitations in physical abilities, older adults with disabilities still appreciate activities that they are capable of doing and provide them with high emotional rewards. Therefore, in addition to encouraging older adults with disabilities to actively engage in activities, it is important to consider the meaning of these activities for them. Enhancing the meaningfulness of activities that older adults engage in may have a greater impact on their QOL, as opposed to simply promoting their activity performance or engagement. The results of this study provide significant insights for professionals working with older adults with disabilities, particularly in the field of occupational therapy. As noted by Leibold et al. [17], it is crucial to incorporate the subjective meaning of activities into client-centered assessments to understand what is meaningful for older individuals and how to effectively support their engagement in activities that align with their values or sense of meaning.

In line with the second hypothesis, the findings confirmed that higher levels of T1 IADL performance were associated with better T2 QOL, providing support for the notion that improving IADL performance among older adults with disabilities can enhance their QOL. Furthermore, this positive effect on QOL persisted up to two years later. This study further confirms that better T1 IADL performance promotes T2 QOL through enhanced T1 IADL performance, and increased IADL meaning at both T1 and T2. These results suggest that both T1 and T2 IADL meaning plays a crucial role during the course of disability, with higher meaning in the earlier stages leading to higher meaning and ultimately higher QOL in the later stages. Additionally, the relationship among IADL performance, IADL meaning, and QOL has been underexplored in previous research. Akaida et al. [9] have noted that disability can limit older adults’ ability to perform daily activities and can also reduce their enjoyment or satisfaction of participating in family life, domestic activities or taking care of their own basic needs. Build upon existing literature, this study provides additional evidence that enabling older adults with disabilities to remain living at home and engaging in activities that they are still able to perform, even if they are basic daily activities, can have a significant benefit on their current and future QOL.

Regarding social activities, this study found that higher levels of social activity performance at T1 were associated with better T2 QOL through two mechanisms: increased social activity performance at T1, and increased social activity meaning at both T1 and T2. The results of this study are consistent with Levasseur et al. [20] and support that social activities are more fulfilling for older adults with disabilities, and they place greater value on such activities than daily activities, making them one of the best predictors of QOL for older adults. Additionally, the findings of this study reveal that T1 and T2 social activity performance and meaning, as well as T1 QOL, explain 41.5% of the variation in T2 QOL in older adults with disabilities, which is greater than that of IADL and exceeds the findings of prior research [20], highlighting the importance of engaging in meaningful social activities for older adults with disabilities. The results thus confirms the third research hypothesis, suggesting that in contrast to IADL, participation in meaningful social activities can be related to older adults with disabilities’ QOL by enhancing their feelings of relatedness [9]. Thus, the engagement in social activities should be considered a choice of lifestyle for older adults living with disabilities, based on their individual abilities, preferences, life habits, expectations, and societal roles. Despite limited physical function, the social activities that older adults with disabilities choose to maintain and engage in can hold significant meaning for them and have high impact on their QOL. The research findings advance Activity Theory and highlight the importance of social activities for improving the QOL of older individuals with physical disabilities in Taiwan. However, given that the unmet participation needs of older adults with physical disabilities are primarily associated with social activities, such as leisure, community engagement, and interpersonal relationships, and considering that their daily activity needs are mostly fulfilled [30], and reviewing current nonpharmacological interventions to mitigate disability in older adults, interventions focused on enhancing meaningful social interactions and social engagement are not commonly employed methods [31]. The study’s findings provide valuable inspiration for professionals working with older persons with disabilities, particularly occupational therapists, to develop meaningful interventions. As Western cultures prioritize independence [32], developing occupational therapy interventions that facilitate social activities and also take into account clients’ values within their cultural, social, physical, and economic context, as well as creating an activity participation-friendly environment, could be particularly beneficial for improving the QOL of older adults with disabilities in a more collective culture, such as Taiwan. In Taiwan, loosening the restrictions on transportation subsidies for long-term care service recipients to various social activity venues, rather than just limiting it to medical purposes, could be a potential starting point. Also, activity meaning can be incorporated into disability and long-term care need assessments. In this way, the program delivery and intervention design for older individuals with disabilities can be tailored to include their needs, preferences, and the accessibility of social activities.

This study employs the SST to guide data collection and analysis, enabling researchers to explicate how activity meaning impacts the link between activity performance and QOL. Moreover, longitudinal data collected in a two-year interval were used to clarify the pathways by which T1 performance affected T2 QOL, as well as the intervening role of T1 and T2 activity meaning. Lastly, by differentiating IADL and social activities, this study investigates the distinctive effects of two types of activity meanings on the association between activity performance and QOL. The results highlight that despite irreversible disability or health decline in later life, the QOL of older individuals can still be sustained or enhanced through engaging in highly meaningful IADL or social activities. In other words, the subjective meaning attached to activities has the potential to compensate for the decline in activity performance, allowing for the preservation of a positive QOL outcome. However, there are a few limitations that need to be addressed in this study. First, the generalizability of the results may be limited as the majority of participants were users of formal services, including home and community-based long-term care or community senior services, with only 10% of respondents not receiving any services. Second, this study utilizes an individual’s ability to perform IADL or social activities as an indicator of activity performance, reflecting the level of assistance required for engagement in various activities. Alternative approaches to access activity performance, such as time spent, can be incorporated as measures of activity performance. Third, based on the results of the EFA analysis, this study only examined the impact of IADL and social activity performance and meaning on the QOL of older adults with disabilities. Future studies are advised to include a broader range of activity types and categories. Fourth, the T2 survey was conducted between April and July 2020, which was at the onset of the COVID-19 pandemic. The pandemic’s preventive measures and social distancing policies may have restricted participants’ opportunities for engagement in activities and also impacted their QOL.

## Conclusions

In summary, this study supports the SST and suggests that IADL or social activities can be ways to promote QOL. Enabling persons with limitations to participate in various types of meaningful activities may sustain or even improve their QOL, despite the irreversible trend of disability. Additionally, IADL and social activities satisfy distinct psychological needs of older adults with disabilities and provide unique contributions to their QOL. When planning service programs, both type of activities should be considered simultaneously to maximize the benefits of activity participation for the QOL of older adults with disabilities. In addition to activity performance, special attention should also be given to both IADL and social activity meaning. Understanding the underlying meaning of activity is crucial in enhancing the connection between activity performance and the QOL of older adults with disabilities in the short and long run. To promote the development of programs that can enhance the current and future QOL of older adults with disabilities, professionals must be sensitive to and support their preferences, preferred lifestyles, and expected social roles, and prioritize their engagement in activities that are most meaningful to them.

## Data Availability

The data is not accessible to the public, but it can be obtained from the corresponding author upon a reasonable inquiry.

## Abbreviations

ADL: Activities of Daily Living
SST: Social-emotional selectivity theory
QOL: Quality of Life
IADL: Instrumental Activities of Daily Living
WHO: World Health Organization’s
